# Linking the *FTO* obesity rs1421085 variant circuitry to cellular, metabolic, and organismal phenotypes in vivo

**DOI:** 10.1126/sciadv.abg0108

**Published:** 2021-07-21

**Authors:** Samantha Laber, Sara Forcisi, Liz Bentley, Julia Petzold, Franco Moritz, Kirill S. Smirnov, Loubna Al Sadat, Iain Williamson, Sophie Strobel, Thomas Agnew, Shahana Sengupta, Tom Nicol, Harald Grallert, Margit Heier, Julius Honecker, Joffrey Mianne, Lydia Teboul, Rebecca Dumbell, Helen Long, Michelle Simon, Cecilia Lindgren, Wendy A. Bickmore, Hans Hauner, Philippe Schmitt-Kopplin, Melina Claussnitzer, Roger D. Cox

**Affiliations:** 1 Mammalian Genetics Unit, MRC Harwell Institute, Oxfordshire OX11 0RD, UK.; 2 Department of Physiology, Anatomy and Genetics, University of Oxford, Oxford, UK.; 3 Big Data Institute, Li Ka Shing Centre for Health Information and Discovery, University of Oxford, Oxford OX3 7FZ, UK.; 4 Broad Institute of MIT and Harvard, Cambridge, MA, USA.; 5 Beth Israel Deaconess Medical Center, Harvard Medical School, Boston, MA, USA.; 6 Research Unit Analytical BioGeoChemistry, Helmholtz Zentrum München, Neuherberg, Germany.; 7 German Center for Diabetes Research (DZD), Neuherberg, Germany.; 8 Institute of Nutritional Medicine, Klinikum rechts der Isar, Technical University of Munich, Munich, Germany.; 9 Else Kröner-Fresenius-Centre for Nutritional Medicine, Klinikum rechts der Isar, Technical University of Munich, Munich, Germany.; 10 MRC Human Genetics Unit, MRC Institute of Genetics and Molecular Medicine, The University of Edinburgh, Edinburgh, UK.; 11 Research Unit of Molecular Epidemiology, Institute of Epidemiology, Helmholtz Center Munich, Germany.; 12 KORA Study Center Augsburg, University Hospital of Augsburg, Augsburg, Germany.; 13 Institute of Epidemiology, Helmholtz Zentrum München, Munich, Germany.; 14 Mary Lyon Centre, MRC Harwell Institute, Oxfordshire, UK.; 15 Nuffield Department of Medicine, University of Oxford, Henry Wellcome Building for Molecular Physiology, Old Road Campus, Headington, Oxford OX3 7BN, UK.; 16 Wellcome Centre for Human Genetics, University of Oxford, Oxford OX3 7BN, UK.; 17 Program in Medical and Population Genetics, Broad Institute, Cambridge, MA 02142, USA.; 18 National Institute for Health Research Oxford Biomedical Research Centre, Oxford University Hospitals NHS Foundation Trust, John Radcliffe Hospital, Oxford OX3 9DU, UK.; 19 Analytical Food Chemistry, Technical University of Munich, Freising, Germany.; 20 Institute for Aging Research, Hebrew SeniorLife and Harvard Medical School, Boston, MA, USA.

## Abstract

Variants in FTO have the strongest association with obesity; however, it is still unclear how those noncoding variants mechanistically affect whole-body physiology. We engineered a deletion of the rs1421085 conserved cis-regulatory module (CRM) in mice and confirmed in vivo that the CRM modulates *Irx3* and *Irx5* gene expression and mitochondrial function in adipocytes. The CRM affects molecular and cellular phenotypes in an adipose depot–dependent manner and affects organismal phenotypes that are relevant for obesity, including decreased high-fat diet–induced weight gain, decreased whole-body fat mass, and decreased skin fat thickness. Last, we connected the CRM to a genetically determined effect on steroid patterns in males that was dependent on nutritional challenge and conserved across mice and humans. Together, our data establish cross-species conservation of the rs1421085 regulatory circuitry at the molecular, cellular, metabolic, and organismal level, revealing previously unknown contextual dependence of the variant’s action.

## Teaser

The obesity-linked FTO regulatory circuitry shows cross-species molecular, cellular, metabolic, and organismal conservation.

## INTRODUCTION

The *FTO* locus has been reproducibly associated with body mass index (BMI) in humans across diverse ethnicities (*1*–*3*). Like the vast majority of trait-associated variants identified by genome-wide efforts, BMI-associated variants at the *FTO* locus map to the noncoding genome, which is still poorly characterized. In addition, genome-wide association study (GWAS) loci characteristically contain dozens of statistically significant variants in high linkage disequilibrium (LD), with regulatory variants often acting at distances up to a megabase (Mb), thus implicating multiple potential effector genes. These confounding factors pose a major challenge when translating genetic associations to mechanisms and therapeutic hypotheses. It has recently been shown that *FTO* intronic regions form functional connections to *IRX3* and *IRX5* (*4*–*6*), an Mb away, suggesting that variants within *FTO* could also affect gene regulatory mechanisms at a long distance. The risk allele at the rs1421085 variant in the *FTO* locus has been connected to cellular consequences in adipocytes and to cellular phenotypes that are relevant for obesity (*4*). Furthermore, multiple variants and tissues, including both adipose and brain, have been implicated in mediating risk at the *FTO* locus, pointing toward multiorgan and multivariant mechanisms (*4*, *6*–*9*).

To date, it is unclear whether rs1421085—independently of correlated neighboring variants—affects an organismal or metabolic phenotype. Because gene expression is context specific, variants often depend on specific in vivo stimuli, such as nutritional challenges, to exert their individual effects (*10*). Model organisms have the potential to allow investigation of the spatiotemporal functional effects of variants and their target genes in relevant cell types and tissues in the whole-body context, linking genetic variation with in vivo metabolic physiology. Although the potential for modeling human regulatory variation in mice strongly depends on the conservation of functional elements between human and mouse, there are many good examples of modeling human enhancers and their function in the mouse, such as the recent mechanistic analysis of the distal enhancer at a human autoimmune and allergic disease risk locus (*11q13.5*), involved in immune regulatory T (T_reg_) cell function in the suppression of colitis (*11*). Noncoding regions of the *FTO-IRX* locus show a notable degree of evolutionary sequence conservation between mouse and human that places them among the top 2% of comparably sized genomic regions (*6*). Furthermore, studies comparing the human regulatory landscape to their murine ortholog have yielded new insight into mechanisms involved in diabetes and obesity, and cross-species conservation-based computational models have successfully been exploited in prioritizing functional regulatory elements that associate with metabolic diseases (*8*, *12*, *13*).

The aim of this study was to recapitulate the *FTO* rs1421085 regulatory circuitry at the whole organismal level using a mouse model that harbors a mutation at the murine orthologous region flanking the human rs1421085 C/T variant. Building on conservation patterns of the regulatory landscape between human and mouse, we confirm previous results reporting *Irx3* and *Irx5* to be target genes of the rs1421085 cis-regulatory module (CRM) in adipocyte progenitors (*4*). In addition, we also reveal a complex context-dependent effect on *Irx3* and *Irx5* target gene regulation, on fat mass and skin fat thickness, and on metabolic consequences under high-fat diet (HFD) conditions. For the latter, we test the effects of the rs1421085 circuitry on metabolic murine phenotypes under HFD conditions using untargeted, ultrahigh-resolution metabolome analysis and show an rs1421085-specific disruption of steroidal metabolic homeostasis. We recover an rs1421085 steroid metabotype (*14*) (metabolic phenotype) in human blood plasma samples following an oral glucose tolerance test (OGTT) in 379 healthy, normal-weight individuals.

## RESULTS

### rs1421085 is within a regulatory region conserved across human and mouse

**Fig. 1:**
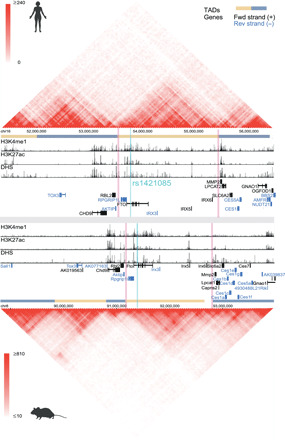
Evolutionary conservation of the chromatin landscape between human and mouse at the region surrounding the FTO/Fto locus. Hi-C data from human H1-ESC (top track) (*16*) and mouse embryonic stem cell (mESC) (bottom track) (*74*) are visualized in the 3D Genome Browser (http://3dgenome.org) (*15*). The heatmap values on a color scale correspond to the number of times that reads in two 40-kb bins were sequenced together (red, strong interaction; white, weak interaction). TADs are indicated at the base of the heatmaps, and the red vertical lines indicate the boundaries of the TAD containing the BMI-associated region in FTO. Histone modification signals (H3K27ac and H3K4me1) for human (*75*) and mouse (*76*) preadipocytes as well as DNase I hypersensitive sites (DHS) peaks for human (*67*) and mouse (*55*) adipose were overlapped and visualized using the 3D Genome Browser (http://3dgenome.org) (*15*). The blue vertical line indicates the position of BMI-associated variant rs1421085 and its orthologous site in mouse.

To develop a murine model system to recapitulate and validate the rs1421085 regulatory circuitry at the organismal level, we first assessed the evolutionary conservation of chromatin structure across humans and mice at the *FTO/Fto* locus. In human adipocyte progenitor cells, the BMI association signal in *FTO*, and more specifically the regulatory variant rs1421085, is within a region decorated by enhancer-associated histone marks (H3K4me1 and H3K27ac; Fig. 1). Cross-species analysis revealed that the peaks for histone modifications associated with regulatory activity in human preadipocytes were also present in mouse preadipocytes [Fig. 1 and fig. S1 (*FTO/Fto* gene zoomed inset)], suggesting a functionally conserved regulatory region surrounding rs1421085.

To test whether the physical environment of proximal and distal target genes are conserved across human and mouse at the *FTO* locus, we examined HiC data surrounding *FTO/Fto*. In human and mouse embryonic stem cells (ESCs) (*15*), we observed topologically associated domains (TADs) (*16*, *17*) that encompassed the same five candidate target genes *Rpgrip1l*, *Fto*, *Irx3*, *Irx5*, and *Irx6* in both human and mouse (Fig. 1). Last, we performed sequence-level analysis of the rs1421085 surrounding region and observed that transcription factor binding site (TFBS) motifs are well conserved at the nucleotide level, including the ARID motif, which was previously shown to be disrupted by rs1421085 (*4*). Together, these data suggest that rs1421085, and more generally the human *FTO* risk locus, localizes within a regulatory element that is conserved at the sequence and chromatin structure level.

### Deletion of the conserved rs1421085 CRM in mice affects body composition

**Fig. 2:**
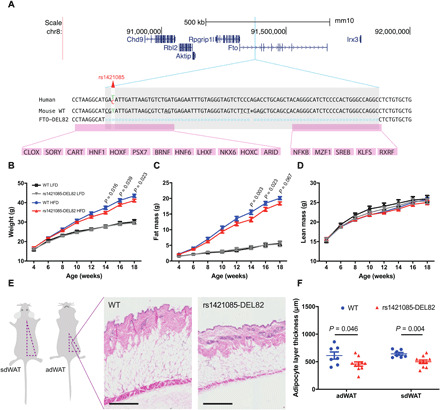
rs1421085-DEL82 results in an adipose depot–specific metabolic phenotype in mouse. (**A**) Schematic of the intron-1 orthologous rs1421085 region in mouse and the 82-nt deletion highlighted in gray. CRMs of TFBSs, conserved in 80% of human, mouse, rhesus, chimp, rabbit, rat, horse, dog, cow, and opossum species, were identified by scanning (+4/−8-nt windows) using Genomatix (Munich, Germany) and are highlighted in pink. (**B** to **D**) Body weight (B), fat mass (C), and lean mass (D) were assessed in WT and rs1421085-DEL82 mice on LFD or HFD in the same animals over a time course. Numbers for each group are male WT HFD (*n* = 17), rs1421085-DEL82 HFD (*n* = 17), male WT LFD (*n* = 13), and rs1421085-DEL82 LFD (*n* = 15). Statistical significance was determined using two-way repeated-measures analysis of variance (ANOVA) and Bonferroni’s multiple comparisons test adjustment. (**E** and **F**) Skin was excised from mice on HFD at 6 months of age for histological processing. (E) Representative histological images of dermal WAT (dWAT). (F) Relative dermal adipocyte layer (dWAT) thickness for skin samples obtained from ventral/abdominal area (adWAT) and the dorsal area/back (sdWAT) calculated from histological assay. Statistical significance was determined using multiple Student’s *t* tests. All data are expressed as means ± SEM.

To dissect the mechanism and physiological role of rs1421085, we used CRISPR/Cas9 genome editing (*18*) to delete the rs1421085 orthologous CRM (rs1421085-DEL82) in the mouse. Specifically, rs1421085-DEL82 harbors a homozygous mutation in the orthologous region between human and mouse, deleting 2 nucleotides (nt) upstream and 79 nt downstream of rs1421085 (Fig. 2A), spanning the ARID motif. None of the other correlated variants of the *FTO* risk haplotype localizes within these 82 nt. To test whether rs1421085-DEL82 affects whole-body composition, we examined mutants versus controls under HFD and low-fat diet (LFD) conditions. We observed that homozygous male rs1421085-DEL82 mice on an HFD showed a subtle reduction in body weight and fat mass from 14 weeks of age (Fig. 2, B and C), which was statistically significant between 14 and 18 weeks of age for body weight (*P* < 0.05; Fig. 2B) and 14 and 16 weeks of age (*P* < 0.05; Fig. 2C) for fat mass. Lean mass was not altered between genotypes (Fig. 2D). Heterozygous rs1421085-DEL82 had an intermediate effect on body weight (fig. S2A) and fat mass gain (fig. S2B) on HFD compared to homozygous alleles, suggesting a dose-dependent effect of the rs1421085-DEL82 allele on body weight regulation and obesity development in male animals. Mice on an LFD did not present with altered body weight or fat mass (Fig. 2, B and C). The rs1421085-DEL82 mutation did not have a detectable effect on body weight or fat mass in female animals (fig. S3, A to C, E and F), and rs1421085-DEL82 had no effect on glucose tolerance in any of the groups (figs. S2, C and D, and S3D).

To further investigate the effect on obesity-related phenotypes, we examined the effect of rs1421085-DEL82 on subcutaneous dermal adipose tissue (sc-dWAT). We quantified sc-dWAT by measuring adipose layer thickness at two anatomical locations, namely, in the ventral-abdominal area and the dorsal area. Subcutaneous adipose tissue below the skin is the major site of fat deposition, accounting for approximately 50% of total body fat mass in mice (*19*). The data revealed that the subcutaneous adipose layer thickness was reduced by 35% at the ventral-abdominal site (*P* = 0.046) and 32% at the dorsal site (*P* = 0.005) in homozygous rs1421085-DEL82 relative to their homozygous controls (Fig. 2, E and F), which was in line with the reduction in body weight and overall fat mass measured by echo magnetic resonance imaging (echoMRI).

### rs1421085-DEL82 is within a regulatory element for *Irx3* and *Irx5* in mouse adipocyte progenitors

To identify the target genes under the genetic control of the rs1421085 CRM, we examined gene expression levels of five candidate target genes contained within the TAD, namely, *Rpgrip1l*, *Fto*, *Irx3*, *Irx5*, and *Irx6*. Obesogenic WAT expansion is mainly regulated by hypothalamic signals in the brain and WAT-intrinsic mechanisms (*20*), and both tissues have been previously implicated in the *FTO* obesity risk locus (*2*, *4*, *6*, *7*). We tested target gene expression in mouse adipose tissue (mostly consisting of mature adipocytes) derived from subcutaneous inguinal WAT (sc-iWAT; a proxy for gluteofemoral adipose tissue in human), visceral perigonadal WAT (vc-gWAT; a proxy for visceral adipose tissue in human), isolated adipocyte progenitors from both these adipose depots, and the hypothalamus.

**Fig. 3:**
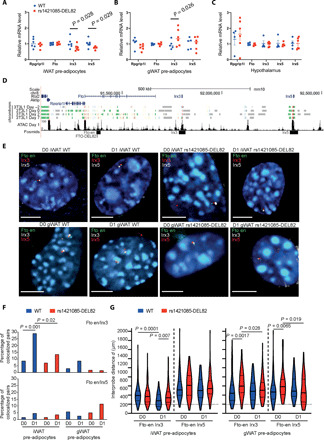
rs1421085 CRM has an adipose depot–specific effect on Irx3 and Irx5 expression in adipocytes. (**A** to **C**) *Rpgrip1l*, *Fto*, *Irx3*, *Irx5*, and *Irx6* gene expression (qPCR) normalized to *Canx* in iWAT (A) and gWAT (B) preadipocytes and in hypothalamus (C) at 6 to 8 weeks. Statistical analysis using Student’s *t* test; means ± SEM. (**D**) Location of genes in 1.6-Mb mouse genomic locus. Tracks 4 to 7 ChromHMM (*77*) 3T3-L1 preadipocyte annotations (*75*). Track 8 ATAC-seq in iWAT preadipocytes at day 1 adipogenic induction. Bottom track position of FISH fosmids. (**E**) Representative FISH nuclei images from iWAT- and gWAT-derived male WT and rs1421085-DEL82 undifferentiated proliferating (D0) or 1 day post-adipogenic stimulation (D1) primary preadipocytes (*n* = 3 animals each). Fluorescent probes for rs1421085-en, *Irx3*, and *Irx5*. Probe distances measured in iWAT D0 WT *n* = 71, rs1421085-DEL82 *n* = 70; iWAT D1 WT *n* = 90, rs1421085-DEL82 *n* = 90; gWAT D0 WT *n* = 71, rs1421085-DEL82 *n* = 62; gWAT D1 WT *n* = 82, rs1421085-DEL82 *n* = 70 nuclei. (**F**) Probe proximity (percentage of colocalized pairs, *d* < 200 nm). Statistical analysis using Fisher’s exact two-tailed tests. (**G**) Violin plots (median and interquartile range) of interprobe distances (nm) between different probe combinations (rs1421085-en/Irx3; Irx3/Irx5; rs1421085-en/Irx5). Horizontal line proportion of alleles that are colocalized <200 nm. Statistical analysis using Mann-Whitney *U* tests.

We observed a decrease of *Irx3* (0.65-fold, *P* = 0.028) and *Irx5* expression (0.71-fold, *P* = 0.029) in sc-iWAT–derived mouse preadipocytes homozygous for rs1421085-DEL82 (Fig. 3A). Heterozygosity for rs1421085-DEL82 had an intermediate effect in *Irx3* (0.81-fold, *P* = 0.0336) and *Irx5* expression levels (0.79-fold, *P* = 0.021) (fig. S4A), suggesting a dose-dependent effect of rs1421085-DEL82 on target gene expression in sc-iWAT preadipocytes.

In contrast to the effect in sc-iWAT progenitors, we observed an increase of *Irx3* (1.5-fold, *P* = 0.026) mRNA levels in vc-gWAT–derived mouse preadipocytes (Fig. 3B). The data further revealed a lack of gene expression changes in female sc-iWAT– or vc-gWAT–derived preadipocytes at any time point (fig. S5, A and B), which was consistent with the lack of an organismal phenotype in female mutant mice (fig. S3). The expression of the *Irx6* gene was below detection limits in both sc-iWAT and vc-gWAT preadipocytes. In addition, we did not observe any effect on *Irx3* and *Irx5* expression in the hypothalamus of male (Fig. 3C) or female animals (fig. S5C), suggesting a cell type–specific effect of rs1421085-DEL82 on gene expression. Last, we show that the rs1421085 CRM deletion did not affect *Fto*, *Rpgrip1l*, and *Irx6* gene expression levels in any tissue examined (Fig. 3, A to C, and fig. S5, A to C). Expression analysis of gWAT and iWAT mature adipose depots from homozygous male rs1421085-DEL82 and control mice revealed that *Irx3* and *Irx5* mRNA levels were not altered (fig. S4B), indicating that target gene expression changes and regulatory activity are specific to early differentiating preadipocytes.

Together, we show that out of the five potential target genes measured in five relevant mouse cell/tissue types, only *Irx3* and *Irx5* were altered in preadipocytes of male rs1421085-DEL82 animals. We observed an opposite direction of target gene expression in vc-gWAT and sc-iWAT adipocyte progenitors, which was in line with fat mass alterations in opposing directions. Differences in gene expression across depots could be due to either a direct effect on regulatory activity or an indirect effect on gene expression mediated by secondary effects independent of the rs1421085 regulatory circuitry.

To examine depot-specific spatial enhancer-promoter proximity between rs1421085 CRM and the target genes *Irx3* and *Irx5*, we performed three-dimensional (3D) fluorescence in situ hybridization (FISH) in iWAT- and gWAT-derived primary adipocyte progenitors of male wild-type (WT) and rs1421085-DEL82 animals. We performed the experiment in actively proliferating cells before confluency and before adipogenesis was induced (D0) and 1 day after induction of differentiation (D1). Fosmids that hybridize to rs1421085-en (enhancer element that overlaps rs1421085), *Irx3*, and *Irx5* were used (Fig. 3, D and E).

In WT sc-iWAT, the *Fto* intron 1 rs1421085 enhancer (rs1421085-en) and *Irx3* promoter probe pairs were significantly more colocalized (from 8 to 29%) and the average distance between them was significantly decreased (*P* = 0.0001), in cells induced for differentiation compared to proliferating cells (D1 versus D0, respectively) (Fig. 3, F and G). In contrast, in rs1421085-DEL82 cells, rs1421085-en and *Irx3* colocalization frequencies were markedly decreased and interprobe distances increased at D1 compared to WT cells (*P* = 0.0072). These data indicate disrupted chromosome conformation upon induction of adipogenesis (Fig. 3G) that may be consistent with a decrease of *Irx3* mRNA levels following rs1421085 CRM deletion in confluent sc-iWAT cells (see above, Fig. 3A). There was less probe colocalization (<5%) and the larger interprobe distances at both time points between rs1421085-en and *Irx5* than was observed with *Irx3*, with no significant differences observed between genotypes (Fig. 3, F and G).

In vc-gWAT, probe colocalization frequencies were lower (<9%) and the interprobe distances were larger for rs1421085-en and *Irx3* promoter in all samples compared to sc-iWAT (Fig. 3, F and G). Nevertheless, we could detect increased distances between rs1421085-en and *Irx3* or *Irx5* in rs1421085-DEL82 compared to WT at D0 (*P* = 0.0017, rs1421085-en/Irx3; *P* = 0.0065, rs1421085-en/Irx5), but not at D1 of differentiation (Fig. 3G). The functional significance of these changes in chromosome conformation is unclear, as there is increased *Irx3* mRNA levels in vc-gWAT from rs1421085-DEL82 mice (Fig. 3B). It is possible that the increase in *Irx3* expression in vc-gWAT could be a secondary effect rather than a direct effect of the genetic locus.

These data together provide evidence for dynamic chromatin conformation changes at the *Fto/Irx3/Irx5* regulatory circuitry in sc-iWAT and vc-gWAT. In particular the strongly increased rs1421085-en and *Irx3* promoter spatial colocalization occurring over D0 to D1 of differentiation is disrupted in sc-iWAT from rs1421085-DEL82 mice.

### rs1421085 CRM controls mitochondrial copy number and mitochondrial function in human and mouse adipose-derived cells

The *FTO* obesity variant circuitry, mediated through *IRX3* and IRX5, has been linked to adipocyte browning in humans and thus to mitochondrial function (*4*). Therefore, to assess the effect of rs1421085-DEL82 on mitochondrial function and activity in WAT, we excised adipose tissues from male mice on an HFD. We show that adipose tissues from rs1421085-DEL82 mice were characterized by a significantly increased number of mitochondria (fig. S6A) with 10.8-fold (*P* = 0.022) and 6.6-fold (*P* = 0.020) increase in vc-gWAT and sc-iWAT, respectively, compared to WT animals. We next examined the expression of marker genes involved in thermogenesis (*Adrb3*, *Pgc1a*, *Ucp1*, *Cox7a*, *Cox7b*, *Prdm16*, *Dio2*, and *Elovl3*) and adipogenesis (*Pparg*, *Cebpa*, *Fabp4*, *Plin1*, and *Fasn*). We found that rs1421085-DEL82 resulted in a significant increase of β-adrenergic receptor 3 (*Adrb3*) expression in gWAT (threefold; *P* < 0.0001) (fig. S6C) and on the browning marker *Elovl3* in sc-iWAT (fig. S6D).

To assess whether gene expression changes in *Adrb3* and *Evolv3* in rs1421085-DEL82 vc-gWAT and sc-iWAT had functional consequences on cellular oxygen consumption rate (OCR), preadipocytes from WT and rs1421085-DEL82 male mice were isolated and differentiated to perform a mitochondrial stress test using the Seahorse Bioflux XF24 Analyser. Under basal conditions, rs1421085-DEL82 did not affect OCR profiles in vc-gWAT– or sc-iWAT–derived preadipocytes (fig. S6B). However, β-adrenergic stimulation resulted in significantly increasing OCR and proton leak (fig. S6B) in gWAT-derived rs1421085-DEL82 preadipocytes, indicative of uncoupled respiration in these cells. In contrast, β-adrenergic stimulation in preadipocytes isolated from rs1421085-DEL82 iWAT had no effect on OCR (fig. S6B). In summary, rs1421085-DEL82 had an effect on mitochondrial traits, affecting slightly different mitochondrial subtraits across the adipose depots.

### Acyl steroids in adipose are under the genetic control of rs1421085-DEL82 in male mice

**Fig. 4:**
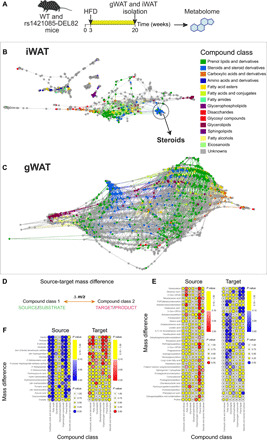
Metabolic profiling reveals a change of steroids in rs1421085-DEL82 male mice under HFD. (**A**) Experimental design. (**B** and **C**) Mass difference analysis (MDiA) to assign unambiguous molecular formulas and map a metabolic mass difference network (MDiN) structure to UHR-MS metabolic features (*78*). Mapping of HMDB database compounds to MDiNs shows clustering of compound classes (legend top right) on the networks of sc-iWAT (B) and vc-gWAT (C) of rs1421085-DEL82 mice. ORA on rs1421085-DEL82 features revealed enrichment of steroids and derivatives in sc-iWAT (*P* = 0.0062) and not in vc-gWAT. However, the topology of the steroid cluster in vc-gWAT implied high chemical diversity demanding more refined analyses. (**D**) A schematic of a generic biotransformation supporting (E) and (F). (**E**) Forward reactions (vertical axis) between down-regulated (consumed) source compound classes and up-regulated (produced) target compound classes on the horizontal axis. Statistical significance of a compound class to be over-represented as a source/target in a reaction was determined using hypergeometric test. (**F**) Backward reactions between up-regulated (produced) source compound classes and down-regulated (consumed) target compound classes. Statistical significance of a compound class to be over-represented as a source/target in a reaction was determined using a hypergeometric test.

Obesity and fat mass–related traits are associated with a dysregulation in blood (*21*) and tissue metabolite levels (*22*). To link the rs1421085 CRM to metabolic changes in adipose, we combined our rs1421085 CRM deletion model on HFD with untargeted metabolome analysis as an agnostic high-dimensional, high-resolution phenotypic readout (Fig. 4A). Untargeted metabolome analysis, which avoids prefiltering on selected compound features, allows to resolve mass to charge ratios (*m/z*) for a wide metabolite space, including metabolites of known and unknown chemical identities. We performed direct-infusion ion-cyclotron-resonance Fourier transform mass spectrometry (DI-FT-ICR MS), known for its ultrahigh resolution and accuracy, to systematically connect rs1421085 genetics with metabolic activity in vc-gWAT and sc-iWAT from rs1421085-DEL82 mice (Fig. 4A). We used mass difference network (MDiN) analysis to assign MS features to high-confidence molecular formulas encompassing known and unknown metabolites (*23*–*27*). Here, “known metabolic features” refers to molecular formulas that have a match in the Human Metabolome database (HMDB) database (*28*). The data revealed that 940 of 2338 differential features matched to known metabolic features in sc-iWAT and 1981 of 7797 features in vc-gWAT when comparing rs1421085-DEL82 mutants with their controls. While any assigned molecular formula can match to multiple isomeric compound structures and annotation is hence putative, molecular formulas are more specific for compound classes, particularly when distinct compound class labels are overrepresented within differential features. To understand the causal connections between fat mass phenotypes (driven by the rs1421085 CRM deletion) and variation in metabolite levels, we performed compound class overrepresentation analysis (ORA) of known metabolic features that differ between the rs1421085-DEL82 mutant and its control littermates (*29*). In sc-iWAT from male mice, we observed a decrease of steroids and their derivatives in rs1421085-DEL82 compared to WT controls (*P* = 0.0062) (Fig. 4B and table S1). We did not observe significant changes in sc-iWAT from females. We found an increase of glycerophospholipids (*P* = 0.0339) and a decrease in organooxygen compounds (*P* = 0.0255) in vc-gWAT from male rs1421085-DEL82 mice compared to their controls (Fig. 4C and table S2). Fatty acyls were strongly overrepresented among both increased features (*P* = 0.0011) and decreased features (*P* = 0.0053), indicating dynamic fatty acyl rearrangements. Steroid compounds did not exhibit significant enrichment within rs1421085-DEL82 vc-gWAT from males when taking all putatively annotated metabolic features into account. However, steroids on the vc-gWAT MDiN (Fig. 4C) showed two topologies: One subset was spread over a wide number of edges, indicating high chemical diversity, and another subset was located in a dense cluster, requiring more refined analyses. We observed a strong decrease of glycerolipids (*P* = 0.00001) in rs1421085-DEL82 female mice (table S3). To evaluate whether steroid homeostasis, which we observed to be altered in sc-iWAT, is perturbed in vc-gWAT as well (and might be missed in the ORA analysis because ORA tests for known metabolites only), we performed mass difference enrichment analysis (MDEA). MDEA screens all pairs of MS features for mass differences of known biochemical transformations. MS feature pairs connected by a biochemical transformation comprise a source node (reactant, substrate) of lower mass and a target node (product) of higher mass, irrespective of annotation status (known or unknown metabolic feature) (Fig. 4D) (*30*, *31*). Last, MDEA tests whether a specific type of transformation, e.g., conjugation to fatty acyls or conjugation to steroid backbones, occurs with MS features of interest. MDEA on metabolic features allows to implicitly model the genotype effect on anabolic (i.e., substrate node is down-regulated, while product node is up-regulated) and catabolic reactions (i.e., substrate node is up-regulated, while product node is down-regulated) (*25*, *32*). We observed a decrease of long fatty acyl steroid compositions in favor of elevated levels of free fatty acids in vc-gWAT from male rs1421085-DEL82 mice compared to their littermate controls. More specifically, we observed forward reactions implicating a consumption of fatty acyls, prenol lipids, and carboxylic acids related to the TCA (tricarboxylic acid cycle), carbohydrate units, and oxidative processes (Fig. 4E and table S4). Reverse reactions imply a production of prenol lipids associated with mass differences of fatty acids and steroids, and a consumption of (i) fatty acyls and glycerolipids associated with mass differences of long unsaturated fatty acids, and (ii) steroids and steroid relatives in association with fatty acyls, which is further substantiated by (iii) consumption of fatty acyls in association with steroids (Fig. 4F and table S5).

Together, these data indicate a stimulation of lipolysis, energy metabolism, and mobilization of fatty acids in adipose from rs1421085-DEL82 compared to their littermates and simultaneously an inhibition of acyl steroid production in rs1421085-DEL82 mice, pointing to a lower adipocyte lipidic esterification and lipogenesis state. These findings suggest that steroid and acyl steroid turnover in sc-iWAT and vc-gWAT is under the genetic control of rs1421085-DEL82 in male mice.

### Connecting the *FTO* rs1421085 genotype to a steroid metabotype in humans

**Fig. 5:**
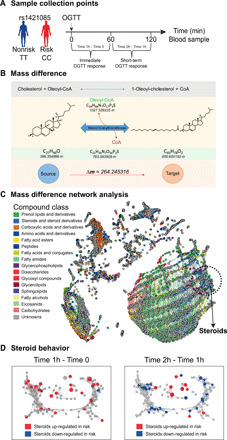
Steroid-related annotations cluster on the MDiN of human plasma metabolic features showing concerted responses to OGTT challenge for the rs1421085 CC risk compared to the nonrisk genotype. (**A**) Experimental design. We examined the immediate OGTT response (the metabolic change between Time 0 and Time 1h) and the short-term OGTT response (the metabolic change between Time 1h and Time 2h). We successively performed MDiN analysis once for the assignment of high-confidence molecular formula (MF) and once for bioinformatic inference, where appropriate (*27*, *31*). (**B**) Example mapping of a biochemical reaction (cholesterol and oleoyl-CoA (coenzyme A) react to give oleoyl-cholesterol and CoA) in the compositional space (i.e., masses and molecular formulas) as the principle of MDiN. The Δ*m* of 264.245316 atomic mass units (amu) is observed as oleic acid is condensed onto a substrate. (**C**) Description of the overall metabolic network by MDiN analysis, colored for HMDB compound class (legend, top left) annotation by exact formula matching or assignment of similar compositions (*79*). (**D**) To appreciate the behavior of this compound class, during the OGTT responses, we extracted this subnetwork from (C). We colored the nodes following up- and down-regulations in the risk class.

Individual predispositions toward diseases can be determined by establishing the effects of genetically determined variants on metabolic phenotypes (metabotypes) (*33*–*38*). To validate the genotype-metabotype finding in rs1421085-DEL82 mice in humans and identify a metabolic pattern in blood plasma that differentiates rs1421085 risk versus nonrisk individuals, we performed high-resolution FT-ICR MS profiling in human blood plasma at baseline and following an OGTT. We chose a two-step discovery-replication scheme. For the discovery stage, we collected blood plasma samples from 50 healthy male individuals from the KORA F4 cohort (*39*) (21 risk allele carriers and 29 nonrisk allele carriers) (table S6). We collected and profiled the samples before (Time 0h), 60 min (Time 1h) after, and 120 min (Time 2h) after an OGTT (*40*). To investigate genotype-dependent effects over the time course of the glucose tolerance test, we modeled the immediate OGTT response (which is the change in metabolic profiles between Time 0 and Time 1h) and the short-term OGTT response (which is the change between Time 1h and Time 2h) (Fig. 5A). Following FT-ICR MS (*41*), we performed MDiN analysis (Fig. 5B) and assigned high-confidence molecular formulas to 8382 metabolic blood plasma features and used multilevel partial least squares discriminant analysis (ML-PLS-DA) (*42*) on both OGTT responses (fig. S7, A to D). The immediate OGTT response was built on a subset of 1045 features (211 known metabolic features), while the short-term OGTT response was built on 1031 features (281 known metabolic features). To identify compound classes that are under the genetic control of the rs1421085 genotype, we performed ORA, which revealed 11 different compound classes significantly contributing to both OGTT responses (fig. S8). These rs1421085-associated compound classes included steroids, amino acids, flavonoids, acyclic alkenes, carbonyl compounds, fatty amides, phenylethylamines, glycerolipids, disaccharides, peptides, and fatty alcohols (Fig. 5C). Notably, the class of steroids and steroid derivatives (table S7) showed the strongest effect among all for both OGTT responses and revealed an opposite behavior (fig. S8), with a notable increase of steroids and their derivatives in risk individuals compared to nonrisk individuals in the immediate OGTT response and a decrease in the short-term OGTT response (Fig. 5D).

**Fig. 6:**
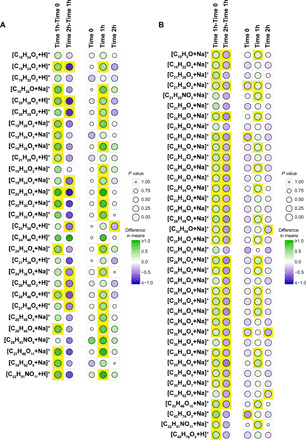
Tile maps describing the main steroid effect in male subjects in response to an OGTT for two independent clinical cohorts. (**A** and **B**) Each panel depicts a set of steroid-associated compositions in CHNO chemical space and the difference in responses of the corresponding compounds to an OGTT between risk-allele carriers versus nonrisk individuals. The left-side columns depict the immediate OGTT response (Time 1h–Time 0) and the short-term OGTT response (Time 2h–Time 1h) by means of a difference in average values of log_2_ fold changes in intensity levels. The right-side columns depict the static differences in average values of log_2_ intensity levels for each time considered. The stronger the green color of the filled circle, the higher the mean value associated with the risk group, compared to the mean value associated with the nonrisk group. In the opposite case, the stronger the blue color of the filled circle, the lower is the mean value. The size of the circles corresponds to a *P* value, from a two-sample Student’s *t* test assuming equal variance (risk and nonrisk). For *P* value lower than 0.05, the corresponding tiles are highlighted in yellow. (A) Tile maps for the KORA F4 cohort. (B) Tile maps for the replication cohort.

When we looked at individual steroid feature effects, we observed a concerted strong up-regulation in the immediate OGTT response and down-regulation in the short-term OGTT response (Fig. 6A, fig. S9A, and table S7). No significant differences were observed at basal condition (Time 0), which is the standard fasted state most commonly sampled for clinical diagnosis of diseases. A substantial amount of features of high significance could be observed in the immediate OGTT response (Time 1h–Time 0), both visually and statistically. Consequently, Time 1h displayed the resulting overall disbalance of steroid compounds. This trend reverted noticeably in the short-term OGTT response (Time 2h–Time 1h). To replicate these results, we recruited 326 healthy subjects from an independent cohort, comprising 243 risk allele carriers (78 males/165 females) and 83 nonrisk allele carriers (22 males/61 females) (table S8). Following FT-ICR MS and matrix generation, for male subjects, we confirmed the strong increase in steroids in the immediate OGTT response (putatively annotated steroids and steroid derivatives, HMDB V3.6) and their decrease in the short-term OGTT response (Fig. 6B, fig. S9B, and table S9). We did not observe a substantial amount of significant rs1421085-dependent features neither at baseline (Fig. 6 and fig. S9) nor in female subjects (fig. S10 and table S10), supporting the context-dependent link of the rs1421085 genotype on the steroidal metabotype.

## DISCUSSION

The *FTO* locus is linked to multiple obesity-related phenotypes through common variant association studies including BMI, fat mass–related traits, and basal metabolic rate, and is considered the strongest genetic risk locus for obesity in humans. We have previously implicated the noncoding variant rs1421085 as a causal variant at the *FTO* obesity risk locus linking rs1421085 to specific effector genes (*IRX3* and *IRX5*) and their cellular functions (mitochondrial energy expenditure following β-adrenergic receptor activation) in human subcutaneous adipocyte progenitors (*4*). Here, we present a murine model system, rs1421085-DEL82, to study the organismal, metabolic, and cellular consequences of the highly conserved CRM flanking the genetic variant rs1421085. We validated previously reported results from human in vitro studies in the rs1421085-DEL82 mouse model and expanded on those results by providing evidence for a context-dependent role of the genetic variant in adipose depots and established an effect on obesity-related whole-body phenotypes. We then used untargeted high-resolution metabolome analysis in adipose and plasma across mice and humans to link the genetic variant to its physiological state and conclude that the rs1421085 genetic variant mediates cross-species conserved changes in steroid patterns following nutritional challenges.

Our data show that deleting the rs1421085-conserved CRM in mice results in decreased *Irx3* and *Irx5* gene expression in iWAT adipocyte progenitors to roughly 70% of WT levels, consistent with regulatory fine-tuning of gene expression by noncoding common genetic variation. We note that the regulatory effect was specific for adipocyte progenitor cells and not visible at the tissue level, which harbor mostly mature differentiated adipocytes. All these results are consistent with previous results from in vitro human subcutaneous adipocyte model systems in vitro, where the rs1421085 variant was edited (fig. S11) (*4*). In those experiments, we established a chain of causation in human preadipocytes in vitro in which the rs1421085 C allele (associated with increased BMI) was an active enhancer resulting in increased *IRX3/5*, decreased mitochondrial function, and increased lipid accumulation. In contrast, the rs1421085 T allele (associated with decreased BMI) was a less active enhancer, resulting in decreased *IRX3/5* expression, increased mitochondrial function, and decreased lipid accumulation. In line with these results, our rs1421085DEL82 mouse model recapitulates the effects of enhancer perturbation (removing the ARID5B TFBS motif rather than changing binding affinity). Consistent with the direction of effect that we observe in human preadipocytes in vitro, the directed perturbation of the rs1421085 flanking region in our rs1421085DEL82 mouse model reduced interaction of this enhancer with its downstream target genes *Irx3* and *Irx5* (see FISH data; Fig. 3, E to G). This loss of enhancer interaction was specific to induced adipocytes (day 1 of differentiation), potentially explaining the reduced *Irx3* and *Irx5* gene expression found in confluent iWAT preadipocytes. In contrast, rs1421085DEL82 had no effect on enhancer interactions with *Irx3* and *Irx5* on day 0 in the FISH experiments, which were actively proliferating cells at this stage. Thus, the effect of rs1421085DEL82 on enhancer interactions and gene expression of target genes seems to depend on the transition from proliferation to induction of the adipogenic program. Last, the rs1421085DEL82 perturbation of the variant flanking regions increased mitochondrial content and resulted in decreased HFD induced body weight gain, fat mass, and skin layer thickness, recapitulating the BMI association in humans.

While we found an expected decreasing effect of rs1421085-DEL82 on *Irx3* and *Irx5* gene expression in subcutaneous adipocyte progenitors from sc-iWAT, we observed an increasing effect on *Irx3* gene expression in adipocyte progenitors from vc-gWAT. Two possible explanations are either that the rs1421085 CRM acts as an enhancer in subcutaneous adipocyte progenitors and a repressor in visceral adipocyte progenitors or, more likely, given the lower vc-gWAT enhancer-promoter colocalization we detect in vc-gWAT compared with iWAT, that *Irx3* up-regulation in gWAT is not a direct consequence of the rs1421085 CRM but rather a secondary compensatory mechanism. While we observed an opposite direction of *Irx3* gene regulation in visceral adipocyte progenitors, we note that our data indicate that cellular and steroidal metabolic changes were consistent across cellular and metabolic phenotypes in both subcutaneous and visceral adipose tissue depots, although we observed stronger genetically determined cellular and metabolic changes in subcutaneous compared to visceral adipose. It would have been interesting to examine expression in sc-dWAT; however, to maintain differentiation capacity in mouse preadipocytes, these cells have to be isolated from mice that are between 4 and 8 weeks of age, and at this age, there is very limited sc-dWAT accessible for dissection. The two depots used in this study are the most readily accessible and relatively well-defined depots, iWAT and gWAT, that are typically used to isolate primary preadipocytes.

Our rs1421085 CRM mouse model indicates a steroid metabotype under the genetic control of rs1421085 CRM in adipose. We confirmed this finding across two independent human cohorts, which provides evidence that the rs1421085 genetically determined steroid metabotype is evolutionarily conserved across mice and humans and detectable in both adipose and plasma. Steroid hormones (including but not limited to sex hormones) are small lipophilic molecules that circulate in the bloodstream controlling many physiological functions including the balance of energy, metabolism, and responses to stress and feeding. Adipocytes are steroidogenic and known to synthesize and metabolize steroid hormones. Mitochondria play important roles in cholesterol homeostasis and the subsequent biosynthesis of steroid hormones, which can, in turn, regulate mitochondrial function. The production of steroids starts at the inner mitochondrial membrane (IMM) with the conversion of cholesterol to pregnenolone by the cytochrome P450 cholesterol side-chain cleavage enzyme (CYP11A1) (*43*). Increasing *IRX3* has been associated with increasing UCP1, which is involved in white-to-beige adipocyte conversion along with mitochondrial fission (*44*, *45*). Mitochondrial fusion was, in turn, reported to be required for steroid biosynthesis (*46*), which links both steroid biosynthesis and thermogenesis to the same event within mitochondrial dynamics (*45*, *47*). Consistently, the rs1421085-DEL82 model showed increased *IRX3* and OCR and inhibited acyl steroid production in vc-gWAT, while decreased *IRX3* and no OCR response to β-adrenergic stimulation were accompanied with increasing steroid levels in sc-iWAT. These findings are consistent with the postprandial steroid pattern responses in blood plasma of human risk allele carriers observed herein.

Large-scale genetic association studies connecting genetic variants to obesity, as assessed by BMI, have implicated the brain as primary tissue of action, and the FTO haplotype has previously been suggested to affect gene expression in the brain (*2*, *6*). In this work, we provide confirmatory evidence that the rs1421085 regulatory element links to context-dependent *Irx3* and *Irx5* gene expression in early differentiating adipocytes and affects body weight–related phenotypes. Noncoding variants can regulate one or more genes across long genomic distances, and the same allele or multiple alleles within a haplotype might have context-specific functions or function in different cell types. Because of the possibility for pleiotropic regulation of multiple genes and multiple cell types at the *FTO* locus, evaluation of multiple variants, candidate genes, and cell types [i.e., adipose and central nervous system (CNS)] is critical to assess causality and to inform how those variants might converge on phenotypes that contribute to the progression of body weight gain. The *FTO* locus associates not only with BMI and fat mass–related traits but also with food preferences (*48*), which could be due to a direct effect of the genetic risk locus on gene expression in the CNS, an indirect effect mediated by a genetically determined metabotype as we described in our study, or a combination of both processes. The observed metabolic features have a high probability to constitute either derivatives of sex hormones or derivatives of progestagens such as cortisol. The mass differences involved with significantly perturbed acyl steroid generation in rs1421085-DEL82 vc-gWAT highlighted aldosterone/cortisol (isomeric) addition and condensation, pregnenolone derivatives, and androgens. Cholesterol, corticosterone, and chenodeoxycholic acid mass differences were not implied in fatty-acyl balance significantly. Cortisol is implied to counteract insulin action and to promote lipogenesis under chronic elevation in blood. A postprandial increase of cortisol derivatives conditioned on reduced mitochondrial activity could promote a vicious circle toward obesity and diabetes. At the same time, our data imply that androgens are affected by rs1421085, and we have found responses to rs1421085 in males only. Sex hormones were previously shown to be implicated in eating behavior, food preferences, and hypothalamic body weight regulation (*49*). The ratio of androgens to estrogens is tightly orchestrating the energy balance regulation, and a shift in this ratio leads to fat accumulation and body weight gain. Our data further indicate a sexual dimorphism of the steroid metabotype, which will need further exploration. Pronounced metabotypic patterns in blood plasma might facilitate risk assessment and planning of preventive strategies, such as paving the way for personalized diets.

Our work stresses the critical role of environment, i.e., nutrition, on the manifestation of a phenotype. We observed a resistance to weight gain conditional on HFD feeding of rs1421085-DEL82 mice, which is consistent with the notion that nutrition is considered to have the strongest environmental effect on obesity risk at the *FTO* locus (*50*, *51*). Our work implicates a strong nutritionally influenced steroid metabotype (metabolic phenotype) under the genetic control of rs1421085 CRM in mice on HFD and in humans following an OGTT. Notably, the link between genotype and metabotype was not significant at baseline and was characterized by a dynamic behavior as revealed by modeling different time points following the OGTT in humans. This is consistent with previous reports, as sex hormones have been shown to underlie a dynamic nutrition-dependent regulation characterized by a postprandial decrease in healthy individuals (*52*).

Modeling noncoding variation in the mouse is challenging given the approximate 90- to 100-million-year divergence between mouse and human. In contrast to coding sequences, which have retained high sequence conservation, regulatory noncoding sequence has undergone a degree of evolutionary rewiring that may confound some cross-species comparisons (*53*). However, there is evidence for significant enrichment of functional GWAS variants in species-conserved TFBSs, which may allow modeling of some functional single-nucleotide polymorphisms (SNPs) in the mouse (*54*). Furthermore, at least 40% of enhancer predictions in the mouse are also predicted to be enhancers in humans, although this is likely an underestimate given the need to match tissue, cell type, and developmental stage to make accurate cross-species comparisons; thus, there will be many opportunities to probe human enhancer function by genetic manipulation in the mouse (*55*). Consequently, for a proportion of species conserved regulatory GWAS variants in enhancers, it will likely be possible to model their physiological function in the mouse, as we demonstrate here. Engineering the rs1421085 CRM allowed us to establish organismal phenotypes under the genetic control of the variant, including HFD-induced weight gain and whole-body fat mass. The in vivo effect of the rs1421085 CRM deletion was more subtle than those reported for global and adipose-specific *Irx3* or *Fto* knockout models (*4*, *6*, *56*–*58*), which is consistent with typically small effect sizes of common genetic variation and, in our study, a small deletion encompassing such a regulatory SNP. We observed a dose-dependent effect of the rs1421085-DEL82 genotype on body weight, fat mass gain, as well as *Irx3* and *Irx5* target gene expression, which is consistent with a dose-dependent effect of the *FTO* risk haplotype on body weight (~3 pounds per allele) (*1*).

Together, our data establish an evolutionary conserved rs1421085 regulatory circuitry, which allowed us to characterize the genetic circuitry across multiple phenotypic scales: (i) adipocyte progenitor as cell types of action; (ii) *Irx3* and *Irx5* as effector genes; (iii) mitochondrial traits as cellular function; and (iv) diet-induced body weight gain and fat mass as organismal phenotypes. We link the rs1421085 circuitry to its genetically determined steroidal metabotype in males, which again was dependent on nutritional challenges.

## MATERIALS AND METHODS

### Metabolome analysis of mouse tissue

#### DI-FT-ICR MS measurements of mouse tissue

sc-iWAT and vc-gWAT were excised at 20 weeks of age and pretreated for the metabotype analysis. Mouse adipose tissue samples (50 mg) were placed in tubes containing ceramic beads (Tissue homogenizing CKMix, 2 ml, P000918-LYSKO-A, Precellys, Bertin Corp., Rockville, MD). One milliliter of cold methanol (−20°C) [LiChrosolv, hypergrade for liquid chromatography–mass spectrometry (LC-MS); Merck KGaA, Darmstadt, Germany] was added to the tissue samples followed by homogenization using the Precellys Evolution Homogenizer (Bertin Corp., Rockville, MD; 5800 rpm, 2 × 15 s, 30-s pause time). The samples were centrifuged (11,292 rcf, 10 min at 4°C), and the supernatants were collected into Eppendorf tubes and stored at −80°C. The extracts, diluted in methanol-water (50:50, v/v) by a factor of 1000, were analyzed in positive electrospray ionization (ESI) mode via DI-FT-ICR MS, using a Bruker SolariX instrument equipped with a 12-T magnet (Bruker Daltonik GmbH, Bremen, Germany). The instrument was externally calibrated by injecting a solution (10 μg/ml) of arginine and observing corresponding peaks with *m/z* values equal to 175.11895 [M+H]^+^, 349.23062 [2M+H]^+^, 523.34230 [3M+H]^+^, and 697.45397 [4M+H]^+^. In the experiment, the flow rate of infusion was set to 120 μl/hour. Four hundred scans, each corresponding to 4 mega words (MWs) in the interval from 147.4 to 1000.0 *m/z*, were acquired and averaged. The ion accumulation time was set to 0.7 s, and the time of flight to the detector was set to 1 ms. The voltages of capillary and spray shields were set to 3800 and −500 V, respectively. The flow rate of nebulizer gas was kept at 2.2 bar, and the drying gas flow rate was set to 4 liters/min (at a temperature of 180°C).

#### Ultrahigh resolution (UHR)–MS data preprocessing

FT-ICR MS spectra were collected and exported using the vendor software’s ftms Control 2.2.0 (Bruker Daltonik, GmbH, Bremen, Germany) and Data Analysis 4.4 (Bruker Daltonik, GmbH, Bremen, Germany), respectively. *m/z* peaks were picked at Signal-to-noise ratio (S/N) ≥ 4, and a minimum intensity of 1.5 × 10^6^ counts was set. Gibbs peaks were removed by Data Analysis automatically. All spectra were exported as tab-separated asc files and loaded into the Kernel Calibrator (*27*). A mouse calibrant list was generated manually after first precalibrating representative mouse tissue spectra against fatty acids, followed by isotopic pattern simulation of the most abundant MS features. Molecular formulas that could be confirmed by means of isotopic fine structure were listed as appropriate. Mouse spectra were then subjected to the Kernel Calibrator. Kernel Calibrator visualizes a mass spectrum’s innate mass error distribution as a density surface, and a calibration function can be optimized under visual inspection until a representative calibration curve is built for each spectrum. The SD of mass error after calibration is generally found to be <100 ppb (parts per billion) at *m/z* < 500 and <300 ppb at *m/z* < 1000. Spectra were further cleaned by removing *m/z* features whose mass defect could not be realized within the acquired *m/z* range given Carbon (C), Hydrogen (H), Nitrogen (N), Oxygen (O), Phosphorus (P), Sulfur (S), Sodium (Na), and *z* = 1.

#### Matrix generation

To perform peak alignment and generate data matrices for vc-gWAT and sc-iWAT cases, all the corresponding mass spectra were subjected to an in-house written matrix generator algorithm that was set to align peaks within a 1-ppm error window.

#### Feature selection

To investigate how the presence or absence of the rs1421085-DEL82 genotype affects WAT (vc-gWAT or sc-iWAT) and to select the most relevant features, we used PLS-DA. Given 7797 features for the vc-gWAT tissue type and 2338 features for the sc-iWAT tissue type, distinct gender-specific PLS-DA models on DEL82 effect were computed. The computation involved an iterative procedure described by van Velzen *et al.* (*42*), where each step represents the selection of a smaller feature subset and building a model on this subset. Before building initial PLS-DA models, the matrices were subjected to a filtering procedure that removed features whose intensity values were present in less than 10% of the included samples. Each missing value was substituted by a minimal intensity value of the corresponding feature multiplied by 0.9. We also applied a logarithmic log_2_ transformation that approximates the distribution of the data to be normal. To assess the quality of PLS-DA models obtained at each step of the aforementioned workflow of van Velzen *et al.* (*42*), we used a sevenfold cross-validation scheme to estimate the values of *Q*^2^ (the cross-validated coefficient of determination *R*^2^). The PLS-DA models were decided to be final if the further model refinement via selecting a smaller subset of features did not improve the values *R*^2^ and *Q*^2^ (*R*^2^ = 0.94 and *Q*^2^ = 0.79 for males from vc-gWAT samples, *R*^2^ = 0.84 and *Q*^2^ = 0.32 for females from vc-gWAT samples, *R*^2^ = 0.98 and *Q*^2^ = 0.88 for males from sc-iWAT samples, and *R*^2^ = 0.97 and *Q*^2^ = 0.82 for females from sc-iWAT samples). All the calculations were done in MATLAB 2018b.

#### MDiN computation and network visualization

##### MDiN reconstruction

MDiNs are reconstructed as published in (*23*–*25*). Briefly, MDiN reconstruction requires a list of mass spectrometric *m/z* values (metabolic features) as nodes and a list of mass differences for the assignment of edges that connect nodes. Mass differences are constructed from known biochemical reactions by subtracting the neutral monoisotopic mass of a substrate from the neutral monoisotopic mass of the corresponding reaction’s product. The mass difference itself is then indicative of the incorporation of the reaction cosubstrate. Expressed in equations: given reaction 1: m(A) + m(B) → m(C), Δmass = m(B) = m(C) − m(A). A pair of nodes xi and xj is connected by an edge corresponding to m(B) if |(m(xj) − m(xi)) − m(B)| < ppm(xi,xj), where ppm(xi,xj) is the mean of the mass measurement errors at m(xj) and m(xi). If the mass difference between the masses of xj and xi is equal to the mass difference m(b) up to a specified error, xi and xj receive an edge corresponding to reaction 1. Each assignment of a mass difference edge to a pair of nodes places a reaction hypothesis on the nodes. Herein, the mass differences from (*25*) were augmented with steroid conjugations (condensations and additions thereof), yielding a set of 487 mass differences. Constructed MDiNs are used for both molecular formula assignment and data visualization.

##### MDiN visualization

Visualizations of MDiNs were performed using Gephi 0.9.2. Networks too dense for visual interpretation are pruned by omitting edges that are not of statistical relevance. MDEA selects mass differences associated with node sets of interest, such as biomarker candidates or compound classes. Herein, MDiNs were visualized using a set of mass differences that is optimal for clustering chemical compound classes as published by Moritz *et al.* (*25*) and Schmitt-Kopplin *et al.* (*59*). The respective mass differences occur within compound classes at a much higher frequency than they do between compound classes. These mass differences separate compound classes optimally, enabling visual detection of biomarker compound class associations on an MDiN.

#### ORA of HMDB compound classes in mouse tissue

Given a number of instances with nonunique properties and a subset from this set, the ORA discloses whether the observed subset, encompassing certain properties, can be obtained by a random sampling rather than some defined process (*29*). We used this notion to understand the general behavior of different compound classes, i.e., whether some compound classes have higher or lower prevalence in mice with the rs1421085-DEL82 genotype. For each of the considered adipocyte tissue types (vc-gWAT or sc-iWAT), the set of all features, used to build an initial PLS-DA model, represented the total number of instances for ORA and therewith the reference distribution of properties. The property of each instance/feature represented a compound class assigned by the HMDB database as well as the sign of the associated regression coefficient. A positive sign of the regression coefficient relates to the higher abundance of the feature in the rs1421085-DEL82 mice, and the negative sign relates to the lower abundance. If no assignment was found, then a generic class, denoting the absence thereof, was given to the feature. The subset of features chosen for the final PLS-DA model represented the subset of instances whose distribution of properties was to be compared to the reference distribution introduced above. For each of the considered classes and its behavior, we used the hypergeometric distribution to estimate the probability (*P* value) whether the corresponding number of features (or less) can be observed in the subset if it was sampled randomly. *P* values for compound class enrichment were computed for the subset of features chosen for the final PLS-DA model as well as for the corresponding subsets with positive and negative regression coefficients. For further investigations, we have considered only those classes whose total number of instances in the reference distribution was higher than 40. All the calculations were done in MATLAB 2018b.

#### ORA of compound classes driven by mass difference analysis

For each of the considered adipocyte tissue types (vc-gWAT or sc-iWAT), the ORA was extended to show which compound classes prevail for each mass difference type selected from the MDEA. Every source and target node was given the corresponding regression coefficient obtained from the initial PLS-DA model. Because source and target nodes correspond to specific features, they were assigned to the respective compound classes obtained from the HMDB database (*28*). If no assignment was present, then a generic class, denoting the absence thereof, was given to the source or target node. For each of the considered mass difference types, the total set of instances referred to all the corresponding mass differences/edges with constituent source and target nodes. In turn, we considered two subsets, where, for each mass difference type, only those mass differences were considered that were characterized (i) by the negative regression coefficients for the source nodes and the positive regression coefficients for the target nodes or (ii) by the positive regression coefficients for the source nodes and the negative regression coefficients for the target nodes. Because a mass difference approximates a real biochemical reaction, these scenarios reflect the transformations happening during this process. Afterward, depending on the use of either the source or target nodes, we performed the ORA to calculate the probability (*P* value) to observe a specific number of features belonging to a certain compound class within the given subset. These calculations were done using the hypergeometric distribution. All the calculations were done in MATLAB 2018b and R using the ggplot2 package.

### KORA F4 cohort: Population characteristics

#### Study samples

Subjects were recruited from the population-based KORA F4 cohort in the region of Augsburg, southern Germany (*39*). The population sample encompassed 50 male individuals of Caucasian origin, aged between 35 and 67 years. The subjects were selected on the basis of the FTO rs1421085 SNP genotype: homozygous controls (TT; *n* = 29), heterozygous risk allele carriers (CT; *n* = 4), and homozygous risk allele carriers (CC; *n* = 17). All individuals underwent OGTT, and the blood samples collected at baseline (fasting state, *t*_0_), 60 min (*t*_1_), and 120 min (*t*_2_) after glucose load were used for the metabolome analysis. Last, a set of 48 subjects sampled at three time points and 2 individuals sampled at two time points was analyzed via DI-FT-ICR MS. Anthropometric and clinical data as well as blood samples were available from a standard 2-hour OGTT (75 g of glucose; Dextro O.G.T., Roche Diagnostics, Mannheim, Germany). Details of the oral metabolic challenge including anthropometric and biochemical measurements, sample collection, and storage are described by Wahl *et al.* (*40*). The study was conducted in accordance with the Declaration of Helsinki. All the participants gave written informed consent, and the Ethics Committee of the Bavarian Medical Association (Bayerische Landesärztekammer) approved the study.

#### Genotyping

KORA F4 samples were genotyped for the SNP rs1421085 with a concordance rate of >99.5% using the MassARRAY system with iPLEX chemistry (Sequenom, USA), as previously described by Holzapfel *et al.* (*60*). All subjects′ FTO rs1421085 genotype was validated by Sanger sequencing. The following primers were used: polymerase chain reaction (PCR) primers: 5′-accatcaaagaggctgttgt-3′ (rs1421085_PCRfwd) and 5′-gcacccattaactcgtcatt-3′ (rs1421085_PCRrev); sequencing primers: 5′-tgtctctaagcccaacaaac-3′ (rs1421085_Seqfwd) and 5′-attgagccatccatcaggtt-3′ (rs1421085_Seqrev).

The PCR was performed with around 50 ng of input genomic DNA in a thermocycler (Biometra, Jena, Germany) as follows: 12 min at 95°C, 50 cycles of 20 s at 95°C, 40 s at 56°C, and 90 s at 72°C, and finally 2 min at 72°C before cooling (*8*).

### Replication cohort: Population characteristics

#### Study samples

For the validation of the findings from the KORA F4 population, we investigated 326 volunteers (males = 100; females = 226) aged between 22 and 75 years who attended a diabetes screening between September 2013 and May 2016 at the Institute for Nutritional Medicine, University Hospital Klinikum rechts der Isar of the Technical University of Munich (prediabetes clinical cohort). The screening addressed individuals with positive family history of type 2 diabetes (first- and/or second-degree relatives), overweight (BMI ≥ 25 kg/m^2^) or obesity (BMI ≥ 30 kg/m^2^), already elevated blood glucose levels in previous examinations, former gestational diabetes, or an increased German Diabetes Risk Score (*61*). The screening was performed in accordance with the Declaration of Helsinki. All the participants gave written informed consent, and the Ethics Committee at the Faculty of Medicine of the Technical University of Munich approved the study. The subjects were selected on the basis of the FTO rs1421085 SNP genotype: homozygous controls (TT; *n* = 83), heterozygous risk allele carriers (CT; *n* = 162), and homozygous risk allele carriers (CC; *n* = 81).

#### Oral metabolic challenge

All individuals underwent a standard 2-hour OGTT (75 g of glucose; Dextro O.G.T., Roche Diagnostics, Mannheim, Germany) starting between 8:00 a.m. and 9:00 a.m. after a 10- to 12-hour overnight fast. Blood samples for standard laboratory measurements and DNA isolation were taken at baseline (fasting values, *t*_0_), 30 min, 60 min (*t*_1_), 90 min, and 120 min (*t*_2_) after glucose load. Samples taken at *t*_0_, *t*_1_, and *t*_2_ were kept for metabolome analyses.

#### Anthropometric and clinical parameters

The standard examination program comprised anthropometric measurements of height (seca 213 portable stadiometer, seca GmbH, Hamburg, Germany), weight, and body composition (Tanita BC-418 MA segmental body composition analyzer, Sindelfingen, Germany), waist and hip circumference, as well as measurement of blood pressure following established protocols.

#### Laboratory measurements

Measurement of standard laboratory/biochemical parameters was performed by SYNLAB clinical laboratory service (Munich, Germany). Hemoglobin A1C (HBA1c), Glutamic Oxaloacetic Transaminase (GOT), Glutamic-Pyruvate Transaminase (GPT), Gamma-Glutamyl Transpeptidase (GGT), cholesterol, Triglyceride (TG), High Density Lipoprotein (HDL), Low Density Lipoprotein (LDL), High-sensitivity C-reactive protein (hsCRP), and Thyroid Stimulating Hormone (TSH) were determined in fasting blood samples, whereas glucose values were determined at every sampling point. Determination of insulin, proinsulin, C-peptide, and nonesterified free fatty acid levels at all sampling points was taken over by IDM (Tübingen Germany).

#### Genotyping

Double-stranded DNA was isolated from EDTA full blood or buffy coat using the DNeasy Blood & Tissue Kit with DNeasy Mini spin-columns (QIAGEN, Hilden, Germany) following the manufacturer’s protocol. The genotyping was partially performed by GENEWIZ UK (Takeley, Essex, UK) and at the Broad Institute of Massachusetts Institute of Technology and Harvard (Cambridge, MA, USA). Genotyping revealed *n* = 243 carriers of the FTO rs1421085 risk allele. In accordance with the discovery study, this group combined/unifies *n* = 81 homozygous (CC) and *n* = 162 heterozygous (CT) carriers. Eighty-three subjects homozygous for the nonrisk allele (TT, *n* = 83) were considered as controls.

### Metabolome analysis of human blood plasma

#### DI-FT-ICR MS measurements of human blood plasma

Plasma samples from a total of 376 subjects (see above for details and ethical approvals), from two independent cohorts, were analyzed via DI-FT-ICR MS (*24*, *41*). Before analyses, the metabolites (from 50 μl of blood plasma) were extracted by C18 solid-phase extraction (SPE) technology, using Omix C18 100 μl tips (Varian) and following the protocol described by Forcisi *et al.* (*24*). The extracts, diluted in methanol by a factor of 50, were analyzed in positive ESI mode via DI-FT-ICR MS, using a Bruker SolariX instrument equipped with a 12-T magnet (Bruker Daltonik GmbH, Bremen, Germany). The instrument was externally calibrated by injecting a solution of arginine (10 μg/ml) and observing corresponding peaks with *m/z* values equal to 175.11895 [M+H]^+^, 349.23062 [2M+H]^+^, 523.34230 [3M+H]^+^, and 697.45397 [4M+H]^+^. In the experiment, the flow rate of infusion was set to 120 μl/hour. Four hundred scans, each corresponding to 4 MWs in the interval from 147.4 to 1000.0 *m/z*, were acquired and averaged. The time of accumulation ion was set to 0.7 s, and the time of flight to the detector was set to 1 ms. The voltages of capillary and spray shields were set to 3800 and −500 V, respectively. The flow rate of nebulizer gas was kept at 2.2 bar, and the drying gas flow rate was set to 4 liters/min (at a temperature of 180°C).

#### UHR-MS data preprocessing

FT-ICR MS spectra were collected and exported using the vendor software ftms Control 2.2.0 (Bruker Daltonik GmbH, Bremen, Germany) and Data Analysis 4.4 (Bruker Daltonik GmbH, Bremen, Germany), respectively. *m/z* peaks were picked at S/N ≥ 4, and a minimum intensity of 1.5 × 10^6^ counts was set. Gibbs peaks were removed by Data Analysis automatically. All spectra were exported as tab-separated asc files and loaded into the Kernel Calibrator (*27*). *m/z* calibration was then performed against an in-house repository of human blood plasma metabolites for both cohorts. Human blood spectra were then subjected to the Kernel Calibrator. Kernel Calibrator visualizes a mass spectrum’s innate mass error distribution as a density surface, and a calibration function can be optimized under visual inspection until a representative calibration curve is built for each spectrum. The SD of mass error after calibration is generally found to be <100 ppb at *m/z* < 500 and <300 ppb at *m/z* < 1000. Spectra were further cleaned by removing *m/z* features whose mass defect could not be realized within the acquired *m/z* range given CHNOPS, Na, and *z* = 1.

#### Matrix generation

To perform peak alignment and generate a data matrix for the KORA F4 cohort, all mass spectra were subjected to an in-house written matrix generator algorithm that was set to align peaks within a 1 ppm error window. Heavy isotopologues were detected and eliminated by searching for *m/z* features at mass spacings corresponding to those of typical heavy isotopes within an error of 3 ppm in conjunction to a correlation coefficient, with their corresponding mono-isotopic peak being *r* ≥ 0.9.

In the replication cohort, to target the steroid-like compounds, the mass spectra were filtered to match the [M+H]^+^ and [M+Na]^+^ adducts of any compound from the HMDB database, belonging to the class of “steroid and steroid derivatives.” The search window was set to 1 ppm. The filtered mass spectra were subjected to an in-house written matrix generator algorithm that was set to align peaks within a 1-ppm error window.

#### MDiN-based molecular formula annotation and network visualization

MDiN-based molecular formula assignment was performed following Tziotis *et al.* (*23*). An MDiN was reconstructed on the data matrix from the KORA F4 cohort, using 0.1-ppm edge error (the error for finding a positively matching *m/z* peak). Molecular formulas to be used as starting points were assigned manually following isotopic fine structure confirmation as described above. Molecular formulas were propagated from assigned starters to nonassigned *m/z* features by propagating the molecular formula difference contained in Mass differences (MDs) to nonassigned neighbors. Newly assigned formulas were tested against the seven golden rules and a modified version of the senior rule. Each newly assigned molecular formula was checked for consistency with all other adjacent *m/z* features of the valid formula. Formulas that did not pass these tests were reset to zero and allowed to be reassigned. *m/z* features with more than 10 unsuccessful assignment events were discarded. The mass error of the resulting molecular formula assignments was found to be on the same scale as reported above for calibration. Unassigned features were discarded. MDiNs were visualized as described in the “MDiN computation and network visualization” section describing the metabolome analysis of mouse data.

#### Feature selection

To investigate how the presence or absence of the FTO rs1421085 SNP genotype in the KORA F4 cohort affects the outcome of the metabolic challenge separated by time intervals as well as to select the most relevant features, we used ML-PLS-DA. The method is capable of handling longitudinal data as described by van Velzen *et al.* (*42*). The original matrix was divided into one containing the samples from the times *t*_0_ and *t*_1_, and another containing the samples from the times *t*_1_ and *t*_2_. Such a division was used to investigate the effect of the metabolic challenge within the corresponding time frame (further referred to as immediate OGTT response and short-term OGTT response, respectively). For feature selection and the construction of final ML-PLS-DA models, we followed the same workflow as in metabolomics analysis of mouse tissue using PLS-DA. All the calculations were done in MATLAB 2018b.

To select the most relevant steroid-like compounds, associated with the effect of *FTO* rs1421085 SNP genotype over OGTT challenge in the replication cohort, we targeted only those features that showed similar behavioral patterns as in the case of the KORA F4 cohort. The missing values in the matrix were substituted by a minimal intensity value of the corresponding feature multiplied by 0.9. We also applied a logarithmic log_2_ transformation that approximates the distribution of the data to be normal. For each gender, the immediate and short-term response matrices were constructed as the difference between the corresponding time points (*t*_1_-*t*_0_ and *t*_2_-*t*_1_, respectively). The criterion to select a feature encompassed all of the following rules: (i) The median value of a feature in the *t*_1_-*t*_0_ matrix should be higher in FTO rs1421085 SNP carriers; (ii) the median value of a feature in the *t*_2_-*t*_1_ matrix should be lower in FTO rs1421085 SNP carriers; and (iii) the *P* value of a two-sample Student’s *t* test (assuming equal variance), describing the effect of the presence of FTO rs1421085 SNP genotype on the immediate or short-term OGTT response, should be lower than 0.1.

#### ORA of HMDB compound classes in humans

Given a number of instances with nonunique properties and a subset from this set, the ORA discloses whether the observed subset can be obtained by a random sampling rather than some defined process, according to the distribution of the properties in the subset (*29*). We used this notion to understand the general behavior of different compound classes, i.e., they increase or decrease during the OGTT responses. The set of all features, used to build an initial ML-PLS-DA model, represented the total number of instances for ORA at each of the considered time frames. The property of each instance/feature represented a compound class assigned by the HMDB database (*28*) as well as the sign of the associated regression coefficient. The latter defines the behavior of the feature: The positive sign relates to the increase, and the negative sign relates to the decrease. If no assignment was found, then a generic class, denoting the absence of thereof, was given to the feature. The subset of features chosen for the final ML-PLS-DA model represented the subset of instances whose distribution of properties was to be compared to the reference distribution introduced above. For each of the considered compound classes and its behavior, we used the hypergeometric distribution to estimate the probability (*P* value) whether the corresponding number of features (or less) can be observed in the subset if it was sampled randomly. Therefore, a low probability indicates that the compound class is strongly associated with its increase or decrease during the OGTT responses. *P* values for compound class enrichment were computed for the subset of features chosen for the final PLS-DA model as well as for the corresponding subsets with positive and negative regression coefficients. All the calculations were done in MATLAB 2018b.

#### Creating boxplots for selected steroids

The selected set of features corresponding to both the KORA F4 and the replication cohorts was represented as five box plots depicting the behavior of these features at specific time points (*t*_0_, *t*_1_, and *t*_2_) as well as during the challenge (*t*_1_-*t*_0_ and *t*_2_-*t*_1_). Before creating boxplots, the original matrix (characterized only by the selected set of features) was divided into three matrices containing the samples from the corresponding time point. They were logarithmically log_2_-transformed, whereas each missing value was imputed by a minimal intensity value of the corresponding feature multiplied by 0.9. To create matrices representing the behavior during the challenge *t*_1_-*t*_0_ and *t*_2_-*t*_1_, we performed the subtraction of the matrices of the corresponding time points. Using only the selected set of features, the boxplots were created using R and its ggplot2 package. For each pair describing the presence or absence of the FTO rs1421085 SNP genotype, we used the two-sample Student’s *t* test with equal variance to calculate the associated *P* value.

### Adipocyte precursor isolation, culture, and differentiation

#### Mouse adipocyte mesenchymal stem cell isolation and differentiation

Mouse primary adipocytes were isolated essentially as previously described by Church *et al.* (*62*). Briefly, individual adipose tissue depots were excised from 6- to 10-week-old mice and placed in phosphate-buffered saline (PBS). Tissues were minced and digested in digestion buffer [sterile Hanks’ balanced salt solution (H8264), collagenase type 2 (0.8 mg/ml) (Worthington Biochemical Corporation, NJ, USA; LS004174), 3% bovine serum albumin (BSA) (with fatty acids)] and incubated in a 37°C water bath for 60 to 75 min with shaking every 10 min by hand. When digestion was complete, tubes were centrifuged for 3 min at 300*g* to separate floating mature adipocytes from the supravascular fraction (SVF), containing adipocyte precursors. The supernatant containing the floating adipocyte fraction was removed, and the cell pellet was resuspended in prewarmed growth medium consisting of Dulbecco’s modified Eagle’s medium (DMEM) GlutaMAX (#10569010 DMEM, high glucose, GlutaMAX Supplement, pyruvate) supplemented with 10% fetal bovine serum (FBS) (Gibco, 10082-147) and 1% penicillin-streptomycin (5000 U/ml) (Gibco, 15070063) and grown at 37°C and 5% CO_2_. The cell solution was subsequently filtered through a 40-μm nylon mesh and plated. Growth medium was replaced the next day, and every 2 days from then.

For differentiation experiments, attached preadipocytes were trypsin-treated using TrypLE Express Enzyme (1X) (Gibco 12605-010), inactivated with growth medium, and counted using either the Scepter 2.0 Handheld Automated Cell Counter (Millipore) with 60-μm tips (Millipore) or the Countess Automated Cell Counter (Thermo Fisher Scientific). Cells (100,000/ml) were plated and grown to confluence. Two days after confluence, preadipocytes were induced to differentiate using induction medium containing growth medium supplemented with 0.5 mM 3-isobutyl-1-methylxathine (IBMX) (Sigma-Aldrich, I5879), 1 μM dexamethasone (Sigma-Aldrich, D2915), and human insulin (5 μg/ml) (Sigma-Aldrich, I9278). On day 4 of differentiation, medium was changed to maintenance medium containing growth medium supplemented with insulin only. Medium was changed every 2 days, and differentiation was completed on days 7 to 9 after adipogenic induction.

#### Human subcutaneous white adipose tissue culture and differentiation

Human primary SVF cells were received from Y.-H. Tseng (Harvard Medical School, Joslin Diabetes Center, One Joslin Place, Boston, MA). The cells were previously isolated and immortalized from human subcutaneous white adipose tissue of a female subject, aged 56 with a BMI of 30.8. Culture and differentiation were performed following the protocol from the originating laboratory as described by Xue *et al.* (*63*). Briefly, preadipocytes were cultured in DMEM GlutaMAX (Gibco, 10569010) supplemented with 10% FBS (Gibco, 10082-147) and 1% penicillin-streptomycin (5000 U/ml) (Gibco, 15070063) at 37°C and 5% CO_2_. For differentiation, cells were treated with 0.25% trypsin (Gibco) and counted using an automatic cell counter, and 100,000 cells per well were seeded in a 12-well plate. Once cells reached confluency, differentiation was induced by adding freshly prepared adipogenic induction medium to cells [DMEM/high glucose supplemented with 10% FBS, 1% penicillin-streptomycin, 33 μM biotin, 0.5 μM human insulin, 17 μM pantothenate, 0.1 μM dexamethasone, 2 nM 3,3′,5-triiodo-l-thyronine (T3), 500 μM IBMX, and 30 μM indomethacin]. Induction medium was replaced with fresh induction medium every 3 days for 24 days, until fully differentiated.

### Oxygen consumption measurements using seahorse Bioflux analyzer

Oxygen Consumption and Bioenergetics Profile was measured using the XF24 extracellular flux analyzer from Seahorse Bioscience. The protocol used in this assay was adapted from Gesta *et al.* (*64*). For this assay, preadipocytes were counted using the Countess (Thermo Fisher Scientific) and 20,000 cells per well were seeded onto seahorse 24-well plates in 100 μl of growth medium and left to adhere overnight. The next morning, 100 μl of growth medium was added to each well. Two or 3 days later (depending on the depot of origin/cell type), cells were induced to differentiate within the seahorse plate following the adipogenic differentiation protocol as described previously. Each cell type was run in 10 replicates, and four wells evenly distributed within the plate were left without cells to use for correction of temperature variation. When the cells were terminally differentiated at days 7 to 9 after adipogenic induction, the assay was performed. For isoproterenol stimulation experiments, cells were treated with 1 μM isoproterenol (Sigma-Aldrich, I6504) overnight (approximately 16 to 20 hours depending on time of assay) before the assay was performed. The evening before the assay, the seahorse XF-24 instrument cartridge was loaded with seahorse calibrant and placed in a CO_2_-free incubator at 37°C overnight. On the day of the assay, cells were washed in XF Assay Media, 2 mM l-glutamine, 2 mM sodium pyruvate, and 10 mM glucose (pH was measured and adjusted to pH 7.4 at 37°C). The Seahorse plate containing the differentiated adipocytes was then incubated for at least 1 hour at 37°C in a CO_2_-free incubator to allow CO_2_ to diffuse out of solution. According to the manufacturer’s protocol, the ports of the seahorse XF-24 analyzer cartridge were then loaded with the following compounds: port A: oligomycin (complex 1 inhibitor); port B: FCCP (carbonyl cyanide *p*-trifluoromethoxyphenylhydrazone; mitochondrial uncoupler); port C: rotenone and antimycin (inhibitors of electron transfer).

Before running the assay, the XF-24 instrument cartridge was calibrated. For total OCR measurements, the minimum OCR reading after rotenone/antimycin A treatment was subtracted from the initial untreated level following the manufacturer’s protocol. To directly measure mitochondrial thermogenesis, uncoupled respiration (proton leak) was measured by subtracting the minimum OCR level after rotenone/antimycin from the minimum level after oligomycin treatment. Oxygen concentrations were measured over time periods of 4 min with 2-min waiting and 2-min mixing. The protocol for a standard bioenergetics profile is composed of basal mitochondrial respiration, adenosine triphosphate (ATP) turnover, proton leak, and mitochondrial respiratory capacity. First, OCR in basal conditions was determined and used to calculate the basal mitochondrial respiration. After this, 2 μM oligomycin was injected from the first port to inhibit ATP synthase, resulting in an accumulation of protons in the mitochondrial intermembrane space and a reduced activity of the electron transport chain (ETC). The resulting decrease in OCR reveals the respiration driving ATP synthesis in the cells, indicating ATP turnover. Residual oxygen consumption capacity can be attributed to the proton leak maintaining a minimal ETC and nonmitochondrial respiration. Next, 2 μM of the mitochondrial uncoupler FCCP was injected, which results in an increase in OCR as the proton gradient across the IMM is dissipated and ETC resumes. This measurement reflects the maximal mitochondrial respiratory capacity. Last, 2 μM rotenone/antimycin A is injected to completely stop ETC activity and the OCR reading at this phase reflects nonmitochondrial respiration.

### Gene expression

#### RNA isolation

Total RNA from all cells and tissues was extracted using TRIzol reagent (Thermo Fisher Scientific, #15596026) following the manufacturer’s instructions. For tissues, 1 ml of TRIzol Reagent was added to 50 to 100 mg of tissue and homogenized using ceramic beads (Precellys) in a Precellys-24 automated homogenizer (Precellys). For RNA extractions, either the RNeasy Mini Kit (Qiagen, #74106) or the Direct-zol RNA MiniPrep Plus Kit (Zymo Research, #R2072) was used. For the RNeasy Mini Kit, 200 μl of chloroform per 1 ml of TRIzol was added and tubes were vigorously shaken for 15 s, followed by 3-min incubation at room temperature. Tubes were then centrifuged at 13,000 rpm for 15 min at 4°C. Following phase separation, the colorless upper phase containing RNA was transferred to a clean tube and an equal volume of 70% EtOH was added. After thorough mixing to precipitate the RNA, the sample was transferred into a RNeasy spin column and consecutive steps were following the manufacturer’s protocol (RNeasy Mini Kit, Qiagen, #74106). For deoxyribonuclease (DNase) digestion, each sample was treated with 10 μl of DNaseI stock plus 70 μl of Buffer RDD (1500 Kunitz units) for 15 min at room temperature (RNase-Free DNase Set, Qiagen, #79254). Contaminant DNA removal was performed between Buffer RW1 washes (350 μl before and 350 μl added just after genomic DNA digestion). Alternatively, 100% EtOH was directly added to the samples in TRIzol following a Direct-zol RNA MiniPrep Plus kit protocol (Zymo Research, #R2072). Purified RNA was quantified using the Epoch Microplate Spectrophotometer (BioTek).

#### cDNA synthesis

Complementary DNA (cDNA) synthesis was performed using the High-Capacity cDNA Reverse Transcription Kit (Thermo Fisher Scientific, #4368814) according to the manufacturer’s protocol.

#### Quantitative reverse transcriptase–PCR

Gene expression was measured using quantitative real-time PCR real-time fluorescence detection. TaqMan Gene Expression Assay reagents and TaqMan FAM dye-labeled probes (Thermo Fisher Scientific) were used to set up appropriate reactions according to the manufacturer’s protocol, using PCR Master Mix (2×) (Thermo Fisher Scientific K0171) and the ABI PRISM 7500 Fast Real-Time PCR System (Applied Biosystems). To determine the most suitable housekeeping gene to use, we performed GeNORM analysis (kit from PrimerDesign) of 12 housekeeping genes in advance. *Canx* was determined as the most stable gene between samples and conditions and used as a housekeeping gene. The following TaqMan probes were used (gene name, TaqMan assay, and TaqMan assay ID): Canx: Mm00500330_m1, Rpgrip1l: Mm00452421_m1, Fto: Mm00488755_m1, Irx3: Mm00500463_m1, Irx5: Mm00502107_m1, Irx6: Mm01253620_m1, Ppargc1a: Mm01208835_m1, Ucp1: Mm01244861_m1, Cox8b: Mm00432648_m1, Prdm16: Mm00712556_m1, Dio2: Mm00515664_m1, Elovl3: Mm00468164_m1, Pparg: Mm00440940_m1, Cebpa: Mm00514283_s1, Fabp4: Mm00445878_m1, Plin1: Mm00558672_m1, Fasn: Mm00662319_m1, Adrb3: Mm02601819_g1, and Cox7a1: Mm00438297_g1.

### Assay for Transposase-Accessible Chromatin (ATAC) sequencing

Assay for Transposase-Accessible Chromatin sequencing (ATAC-seq) was performed by adapting the protocol from Buenrostro *et al.* (*65*). Differentiating cells were lysed directly in the cell culture plate. Lysis buffer was added directly onto cells grown in a 12-well plate. Plates were incubated on ice for 10 min until cells were permeabilized and nuclei were released. Lysis buffer was gently pipetted up and down to wash nuclei off the well and transferred into a chilled 1.5-ml tube to create crude nuclei. Nuclei were spun down at 600*g* for 10 min at 4°C, nuclei pellets were then resuspended in 40 μl of Tagment DNA (TD) Buffer, nuclei were counted using trypan blue, and a volume of 50,000 nuclei was determined. Transposition reaction was performed following a previously published protocol (*65*). Tagmented DNA was PCR-amplified for 11. Quality was assessed using the DNA1000 Chip (Applied Biosystems) and run on a Bioanalyzer (Applied Biosystems). The profiles showed that all libraries had a mean fragment size of ~190 base pairs (bp) and characteristic nucleosome patterning, indicating good quality of the libraries. Libraries were sequenced at the Wellcome Trust Centre for Human Genetics in Oxford on the HiSeq4000 Illumina generating 50 Mio reads/sample, 75-bp paired end. To reduce bias due to PCR amplification of libraries, duplicate reads were removed. Sequencing reads were aligned to mm10. BWA-MEM was used for mapping.

### ATAC-seq statistical evaluation

Trimmed reads were aligned to the mm10 mouse reference genome using Bowtie 2 (*66*). Reads mapping to the mitochondrial genome and alternative contigs were excluded from further analysis. After removing reads with a mapping quality score < 30, peaks were filtered to exclude (*67*, *68*) those with duplicates or that overlapped with a blacklisted region. We used MACS2 (*69*) with “--nomodel --shift -100 --extsize 200 -q 0.05” for the detection of open chromatin. The resulting bed files were converted into coverage tracks (i.e., bigWig format) for visualization and are deposited in the Gene Expression Omnibus (GEO) database under accession number GSE169032.

### 3D FISH

Preadipocytes were isolated as described in the “Adipocyte precursor isolation, culture, and differentiation” section. Adipocytes were plated on Superfrost Plus slides (Thermo Fisher Scientific, REF J1800AMNZ) placed in quadriPERM dishes. Cells were either fixed 1 day after they attached (D0) or induced to differentiate for 2 days (D2). For fixation, cells were washed three times in PBS and fixed in 4% paraformaldehyde for 10 min. After three washes in PBS, cells were permeabilized in 0.5% Triton X for 10 min, followed by three washes in PBS. Slides were then air-dried for approximately 30 min and frozen at −80°C. Slides were then shipped to Iain Williamson (Wendy Bickmore group) at The MRC Human Genetics Unit, Institute of Genetics and Molecular Medicine, University of Edinburgh, where in situ hybridization was performed with the following fosmids (http://bacpacresources.org): (mm9 coordinates) rs1421085-en (WIBR1-0590 M09) chr8: 93911541-93950923, Irx3 (WIBR1-1206E19) chr8: 94307305-94341470, and Irx5 (WIBR1-1060D04) chr8: 94965335-94904882.

Briefly, between 160 and 240 ng of biotin- and digoxigenin-labeled and direct-labeled (Green 496 dUTP, Enzo Life Sciences) fosmid probes were used per slide, with 16 to 24 mg of mouse Cot1 DNA (Invitrogen) and 10 mg of salmon sperm DNA. EtOH was added, and the probe was air-dried. Hybridization mix containing deionized formamide, 20× SSC, 50% dextran sulfate, and Tween 20 was added to the probes for ~1 hour at room temperature. Slides were treated with ribonuclease (RNase) for 1 hour at 37°C in 2× SSC (100 μg/ml) followed by a series of alcohol washes (70, 90, and 100%). Denaturing was performed by heating the samples at 70°C for 5 min and then for 15 min at 80°C before a second series of alcohol washes (70% ice cold), and slides were then left to air dry. The hybridization mix containing the probes was added to the slides, and the probes were hybridized to the target DNA overnight at 37°C. Following a series of washes in 2× SSC (45°C) and 0.1× SSC (60°C), slides were blocked in blocking buffer (4× SSC, 5% Marvel) for ~5 min. The following antibody dilutions were made: rhodamine anti-dig FAB fragments (Roche) 1:20, Texas Red anti-sheep 1:100, biotinylated anti-avidin (Vector) 1:100, and streptavidin Cy5 1:10.

The slides were incubated with antibody in a moistened chamber at 37°C for 30 to 60 min in the following order with 4× SSC/0.1% Tween 20 washes in between: rhodamine anti-dig (Roche, catalog no. 11207750910, lot 35710000), Texas Red anti-sheep (Abcam, catalog no. ab6745, lot GR29419-7)/streptavidin Cy5, biotinylated anti-avidin (Vector, catalog no. BA-0300, lot ZF-0415), and streptavidin Cy5 (Amersham, catalog no. PA45001, lot 17037668). For nuclear staining, slides were treated with 1:1000 dilution of 4′,6-diamidino-2-phenylindole (DAPI) (stock 50 μg/ml) for 5 min before mounting in Vectashield under a coverslip.

### Image analysis

Following procedures previously described by Williamson *et al.* (*70*), slides were imaged using a Photometrics CoolSNAP HQ2 CCD camera and a Zeiss AxioImager A1 fluorescence microscope with a Plan Apochromat 100× 1.4 numerical aperture (NA) objective, a Nikon Intensilight Mercury–based light source (Nikon UK Ltd., Kingston-on-Thames, UK), and either Chroma #89014ET (three-color) or #89000ET (four-color) single excitation and emission filters (Chroma Technology Corp., Rockingham, VT) with the excitation and emission filters installed in prior motorized filter wheels. A piezoelectrically driven objective mount (PIFOC model P-721, Physik Instrumente GmbH & Co., Karlsruhe) was used to control movement in the *z* dimension. Step size for z stacks was set at 0.2 mm. Hardware control, image capture, and analysis were performed using Nikon Nis-Elements software (Nikon UK Ltd., Kingston-on-Thames, UK). Images were deconvolved using a calculated point spread function with the constrained iterative algorithm of Volocity (PerkinElmer Inc., Waltham, MA). Image analysis was carried out using the Quantitation module of Volocity (PerkinElmer Inc., Waltham, MA). For DNA FISH, only alleles with single probe signals were analyzed to eliminate the possibility of measuring sister chromatids.

### Mouse methods

#### Animal husbandry

All animal studies were approved by the Medical Research Council Harwell Institute Animal Welfare and Ethical Review Board, and all procedures were carried out within project license restrictions (PPL 30/2642 and 30/3146) under the UK Animals (Scientific Procedures) Act 1986 and following the ARRIVE guidelines for animal research. Mice were housed according to UK Home Office welfare guidelines in a 12-hour light/dark cycle at a temperature of 21 ± 2°C and humidity of 55 ± 10%. Mice were fed ad libitum and had free access to water (25 ppm chlorine). The research diets used in this study are indicated in the figures. Mice that were used for primary preadipocyte isolation were fed SDS maintenance chow (RM3, 3.6 kcal/g). The high-fat and low-fat research diets used for the phenotyping cohorts were the following matched diets from Research Diets Inc.: D12492 and D122450J, respectively. Cohort size was estimated from previous experiments using the same tests and power calculations using G*Power 2. For the phenotyping cohorts, mice were weaned and randomized onto their respective diets at 3 weeks of age. All in vivo phenotyping was performed blind as was primary cell culture work, with the exception of the selected genotypes used in FISH and Seahorse analysis.

### Genetic mouse model generation

Using CRISPR/Cas9 technology, the mouse models described in this study were generated by the Molecular & Cellular Biology group at MRC Harwell essentially as published by Mianné *et al.* (*71*). The aim was to create a point mutation at the mouse ortholog of human rs1421085, namely, to alter the T to a C, mimicking the human BMI risk allele, and the rs1421085-DEL82 allele was generated in the process as a result of nonhomologous end joining (NHEJ).

#### Design tool and choice of guide

Single-guide RNA (sgRNA) sequences were designed using http://tefor.net/ (*72*) with the guide sequence most proximal to the intended change applied (5′-3′: TAATCAATACGATGCCTT, PAM sequence: AGG). The sgRNA applied is truncated with the intention of increasing specificity for the target site.

#### Donor oligonucleotide template

Donor sequence templates were designed with homology arms of 60 nt in size flanking the intended point mutation. The donor template sequence was ordered as an Ultramer DNA oligonucleotide from IDT (5′-3′: TGCCCTGTGGCTGCAGCTCAGAAGGCTGCCCTACAAATTCTCACTAGACGCTTAATCAATGCGATGCCTTAGGACTCGAACTGCTACCGTAAAATCAATATTACCTTTATTTTAAGTAGCA). The sequences of oligonucleotides, protospacers, and donor DNAs used within the examples presented in this study are shown as follows: reagent name 5′-3′ sequence: sgRNA_#1 sequence TAATCAATACGATGCCTTAGG, sgRNA_#2 sequence AGCGTCTAGTGAGAATTTGTAGG.

#### Pronuclear microinjections of zygotes

Pronuclear microinjection into C57BL/6NTac embryos was performed as described by Gardiner and Teboul (*73*). Cas9 mRNA (Tebu-Bio), sgRNAs, and single-stranded oligo DNA nucleotides (ssODN) were diluted in microinjection buffer [10 mM tris-HCl, 0.1 mM EDTA, and 100 mM NaCl (pH7.5)] and injected using a FemtoJet system (Eppendorf) at concentrations of 200 or 100 ng/μl, 100 or 50 ng/μl each, and 50 or 20 ng/μl, respectively. Injected embryos were reimplanted in CD1 pseudo-pregnant female hosts, which were allowed to litter and rear *F*0 progeny. DNA for genotyping was derived from mouse ear clips.

#### Establishment of colony

Founder animals with evidence of a NHEJ allele were crossed to WT C57BL/6NTac animals. The rs1421085-DEL82 allele was characterized at the F1 generation by PCR and Sanger sequencing using the following primers (5′-3′): Geno_FTO_rs_F1, TTCCTGAGCTAGTGTGTGTACC; Geno_FTO_rs_R1, GTCAGATTAAGGTGACGGGC.

#### Genotyping

Samples were genotyped with both WT and loss of allele mutant assays together with an internal control for copy counting in a qPCR TaqMan assay. In this assay, there were (i) universal probe and universal primer designed near the CRISPR deletion for both alleles, (ii) a WT-specific primer in the deletion designed for the WT allele, and (iii) a mutant-specific primer that bridges the junction designed for the CRISPR mutant allele.

The following Fto-DEL82 WT1 assay (FAM-labeled) primers were used: Fto-DEL82-Univ-Probe (5 nmol), CAGGAGCCAGATTGTCCACAGCA; Fto-DEL82-WT-R located in deletion (15 nmol), GGCTGCCCTACAAATTCTCACTAG; Fto-DEL82-Univ-F (15 nmol), CAGGCAAAAGCAAAAGGTGACATAC.

The following Fto-DEL82 MUT1 assay (FAM-labeled) primers were used: Fto-DEL82-Univ-Probe (5 nmol), CAGGAGCCAGATTGTCCACAGCA; Fto-DEL82-MUT-R (15 nmol), AGGGTCAGCACAGAGATGC; Fto-DEL82-Univ-F (15 nmol), CAGGCAAAAGCAAAAGGTGACATAC.

See the Supplementary Materials (“Fto-DEL82 qPCR genotyping strategy”) for sequence alignments showing the position of the probe and primers in these assays.

The following *Dot1l* internal control (VIC-labeled) primers were used: primer 1, GCCCCAGCACGACCATT; primer 2, TAGTTGGCATCCTTATGCTTCATC; probe, CCAGCTCTCAAGTCG.

### Mouse phenotyping techniques

#### Body composition

Total body weight was measured every 2 weeks on a scale calibrated to 0.01 g. Body composition measurements were performed every 2 weeks using an echo MRI (Echo-MRI-100, EchoMRI, Texas, USA). The readouts were total fat mass (g), lean mass (g), and free water in live, nonanesthetized mice.

#### Intraperitoneal glucose tolerance test

Intraperitoneal glucose tolerance test was performed in the morning after an overnight (up to 16 hours) fast. Body weight of the animals was measured, and a local anesthetic was administered to the mouse tail (EMLA cream, Eutectic mixture of Local Anaesthetics Lidocaine/Prilocaine, AstraZeneca, UK). To establish the baseline glucose level at time point zero, blood glucose levels were measured using the handheld Alphatrak (Abbott) glucose monitor with Alphatrak strips (Abbott). Subsequently, 2 g of glucose per kilogram of body weight (20% glucose in 0.9% NaCl) was injected intraperitoneally and blood glucose levels were measured at 30, 60, and 120 min after injection. A fresh strip was used for each reading.

#### Retro-orbital bleed

Animals were euthanized by intraperitoneal injection of an overdose of anesthetic (0.2 ml of pentobarbitone) in accordance with Home Office procedures. Once fully anesthetized, a glass capillary was inserted into the anterior corner of the mouse eye to perforate the membrane of the retro-orbital sinus. Blood was collected from the capillary in Lithium-Heparin microvette tubes (CB30, Sarstedt, Nümbrecht, Germany). After cervical dislocation, animals were dissected and tissues were taken for weight, histology, and gene expression analysis.

#### Statistical analysis

Mouse data were analyzed using GraphPad Prism 8 Software and tests as indicated in the figure legends.

### Human research participants

We have complied with all relevant ethical regulations.

## SUPPLEMENTARY MATERIALS

Supplementary material for this article is available at http://advances.sciencemag.org/cgi/content/full/7/30/eabg0108/DC1

## Appendix

View/request a protocol for this paper from *Bio-protocol*.
